# Validation of the brazilian version of the oral health impact profile - Aesthetic questionnaire

**DOI:** 10.1590/0103-6440202204237

**Published:** 2022-03-07

**Authors:** Simone Assayag Hanan, Flávia Cohen-Carneiro, Fernando José Herkrath, Branca Heloisa de Oliveira, Alessandro Dourado Loguércio, Leandro de Moura Martins, Ana Paula Corrêa de Queiroz Herkrath

**Affiliations:** 1 School of Dentistry, Federal University of Amazonas, Manaus, Amazonas, Brazil.; 2 Superior School of Health Sciences, State University of Amazonas, Manaus, Amazonas, Brazil.; 3 Instituto Leônidas e Maria Deane, Oswaldo Cruz Foundation, Manaus, Amazonas, Brazil.; 4 Department of Community and Preventive Dentistry, School of Dentistry, Rio de Janeiro State University, Rio de Janeiro, Rio de Janeiro, Brazil.; 5 School of Dentistry, Department of Restorative Dentistry, State University of Ponta Grossa, Ponta Grossa, Paraná, Brazil.

**Keywords:** Dental esthetics, quality of life, tooth bleaching, questionnaire, validity

## Abstract

The aim of the study was to develop the Brazilian version of the Oral Health Impact Profile - Aesthetic Questionnaire (OHIP-Aes-Braz) and test its psychometric properties. The questionnaire test versions were developed by a panel of experts and a pre-test was conducted in a focus group. Data used for testing its psychometric properties were obtained from a randomized controlled clinical trial on tooth bleaching. Seventy-nine Brazilian adults were included. The questionnaires were applied before tooth bleaching treatment (baseline), one week (T1), and one month after the intervention (T2). Reliability was assessed in terms of internal consistency and stability, while validity was ascertained by criterion and construct validity. The sensitivity to change was assessed comparing the total scores at baseline and T2, using the Wilcoxon test (α = 0.05). Both stability and internal consistency (intra-class correlation coefficient=0.95, Cronbach’s α = 0.92) proved to be adequate. Construct validity was confirmed as the correlation between OHIP-Aes-Braz scores with tooth color satisfaction and self-perceived oral health were in the expected direction. A positive correlation between OHIP-Aes-Braz and OHIP-14 (rs=0.63) and OIDP (rs=0.77) was observed. The instrument was responsive once differences in total scores before and after treatment were statistically significant (p<0.001). The OHIP-Aes-Braz presented good psychometric properties and showed sensitivity to change regarding aesthetics evaluation in Brazilian adults treated with tooth bleaching. A valid and reliable instrument allows a suitable assessment of oral health-related quality of life in Brazilian patients submitted to aesthetics dental interventions.

## Resumo

O objetivo do estudo foi desenvolver a versão brasileira do Oral Health Impact Profile - Aesthetic Questionnaire (OHIP-Aes-Braz) e testar suas propriedades psicométricas. As versões de teste do questionário foram desenvolvidas por um painel de especialistas e um pré-teste foi realizado em um grupo focal. Os dados usados para testar suas propriedades psicométricas foram obtidos a partir de um ensaio clínico randomizado controlado de clareamento dental. Setenta e nove brasileiros adultos foram incluídos. Os questionários foram aplicados antes do tratamento clareador (baseline), uma semana (T1) e um mês após a intervenção (T2). A confiabilidade foi avaliada em termos de consistência interna e estabilidade, enquanto a validade foi avaliada pela validade de critério e de construto. A sensibilidade à mudança foi avaliada pela comparação entre os escores totais no baseline e T2, usando o teste de Wilcoxon ((=0,05). Tanto a estabilidade quanto a consistência interna (coeficiente de correlação intraclasse=0,95, alfa de Cronbach=0,92) mostraram-se adequadas. A validade de construto foi confirmada uma vez que a correlação dos escores do OHIP-Aes-Braz com a satisfação com a cor dos dentes e a autopercepção da saúde bucal foram na direção esperada. Uma correlação positiva entre OHIP-Aes-Braz e OHIP-14 (*r*_*s*_ =0,63) e OIDP (*r*_*s*_ =0,77) foi observada. O instrumento foi responsivo uma vez que as diferenças nos escores totais antes e depois do tratamento foram estatisticamente significantes (p<0,001). O OHIP-Aes-Braz apresentou boas propriedades psicométricas e mostrou sensibilidade a mudanças na avaliação estética em adultos brasileiros submetidos ao clareamento dental. Um instrumento válido e confiável permite uma avaliação adequada da qualidade de vida relacionada à saúde bucal em pacientes brasileiros submetidos a intervenções odontológicas estéticas.

## Introduction

Oral health has traditionally been assessed by clinical criteria, which does not express the subjective impact of oral health problems on individuals’ lives. The shift from the disease-centered concept of health (biomedical model) into a multidimensional theory led to the emergence of the construct health-related quality of life (HRQoL). The understanding that oral health is also related to the individuals’ physical, mental and psychosocial well-being denotes the importance of oral health in people’s quality of life ^1^. Oral health related quality of life (OHRQoL) expresses to what extent oral disorders affect psychosocial functioning and well-being ^2^. Among other features, dental aesthetics can impact OHRQoL, which also explains the growth of cosmetic dentistry ^3^ ^,^ ^4^.

Aesthetics represents a concept based on subjective perception and varies from individual to individual. Thus, dental aesthetic itself, as well as the results of interventions to improve it, are hard to evaluate, especially if aesthetics’ assessment uses professional normative criteria but does not account for the individual’s perspective ^3^. Normative clinical indicators measure biological sequelae, but not the suffering, limitations, and expectations faced by individuals due to a pathological condition. On the other hand, self-reported subjective measures help to understand the patient’s perceptions and needs when making therapeutic decisions and in the evaluation of the results of dental treatments ^5^.

Several instruments have been used to measure OHRQoL. The Oral Health Impact Profile (OHIP) is one of the most used for this purpose. Some authors have shown an association between dental aesthetics and OHRQoL, which makes OHIP suitable for the study of the impacts of aesthetic dental treatments ^3^ ^,^ ^6^ ^,^ ^7^. This instrument originally presents 49 questions (OHIP-49), which limits its use in clinical studies. Thus, shorter versions were developed, including the aesthetics one: the OHIP-Aesthetic (OHIP-Aes). OHIP-Aes was firstly developed by Wong, Cheung and McGrath ^8^ in a Chinese population submitted to tooth bleaching. The instrument derived from the Chinese version of OHIP-49, and was originally published in the English language ^8^. OHIP-Aes seems to be more appropriate than OHIP-49 and other OHIP versions to detect changes involving dental aesthetics, especially in relation to tooth color ^8^ ^,^ ^9^.

Cross-cultural validation of quality-of-life questionnaires are important so that they can be applied in locations and cultural contexts other than that where they were developed, also allowing for comparison of results between countries and different populations ^5^. The OHIP-Aes has already been validated in two languages - English ^8^ and Spanish ^5^, but not in the Portuguese language yet. Studies developed in Chile have used the version of OHIP-Aes translated and validated into Spanish by Núñez et al. ^5^ to assess the impact of tooth whitening on patients’ quality of life ^4^ ^,^ ^10^ ^,^ ^11^ ^,^ ^12^ ^,^ ^13^. The English version has also been used to assess the impact of tooth whitening ^8^.

The aim of this paper was to develop the Portuguese version of OHIP-Aes, through translation and cross-cultural adaptation, as well as to evaluate the psychometric properties (reliability, validity, and responsiveness) of the instrument, in a population of Brazilian adults treated with tooth bleaching.

## Material and methods

### Study design and sample calculation

The study was designed to validate the OHIP-Aes instrument in the Portuguese version (OHIP-Aes-Braz), using a sample of patients undergoing tooth whitening in a controlled, randomized, parallel and triple-blinded clinical trial, approved by the Research Ethics Committee (CAAE number 44564115.4.0000.5020) and registered in the Brazilian Clinical Trials Registry (RBR-9mys6q). The clinical trial objective was to test the efficacy and tooth sensitivity to whitening treatment in adult patients who used desensitizing toothpaste, compared to those who used regular toothpaste. The dentifrice tubes were covered with an opaque band marked with numbered codes to blind the participants to group allocation.

The sample size was calculated considering that the study should have a 5% significance level and 80% power to detect a minimal difference of 3.5 in the overall score of OHIP-Aes between the two treatment groups. Previous studies had suggested that this detectable difference should be at least 3 to 5 units ^4^ ^,^ ^14^. Thus, in order to allow for 15% dropout rate, a sample size of 80 patients (40 per group) was determined.

A total of 79 adult individuals, aged 18 or over (range 18-39), of both sexes participated in the study; after being informed about the study objectives and procedures they signed the informed consent forms and were enrolled. Eligibility criteria were individuals with the six maxillary anterior teeth free of caries, without restorations on the labial surfaces, with superior central incisors color shade A2 or darker (Vita Classical; Vita Zahnfabrik, Bad Säckingen, Germany). The exclusion criteria were individuals who reported previous anterior teeth sensitivity or who presented anterior teeth with enamel cracks, exposed dentin, or severe internal discoloration (e.g., tetracycline stains, fluorosis, etc.), individuals with active periodontal disease, pregnant or lactating women, and patients using any anti-inflammatory, analgesics or antioxidant medicines.

### Dental whitening procedures, evaluation, and application of questionnaires

Participants answered OHIP-Aes questionnaire before being submitted to tooth whitening. The instrument was applied in duplicate at this stage, with a one-week interval between assessments (time 0 = baseline and time 0.1 = one week after baseline, before intervention). The questionnaire was self-administered, always under the supervision of a researcher, who was trained to help the participants to answer the questionnaire with standardized explanations, when asked to. Tooth whitening was performed in two sessions with the in-office technique, using 35% hydrogen peroxide (Whiteness HP Maxx, FGM Produtos Odontológicos, Joinville, SC, Brazil) in three 15-minute applications per session, with an interval of one week between sessions, following the manufacturer’ recommendations.

The color evaluation was performed using a Vita classical (Vita Zahnfabrik, Dad Säckingen, Germany) value-oriented shade guide, by two calibrated operators who were blinded to the allocation assignment. The data was registered at the beginning (time 0) and one month (time 2) after the intervention. A variation of Vita Classical units between both times (() was used to measure the color change. The intensity of tooth sensitivity was also registered using a Visual Analogue Scale.

**Figure 1:**
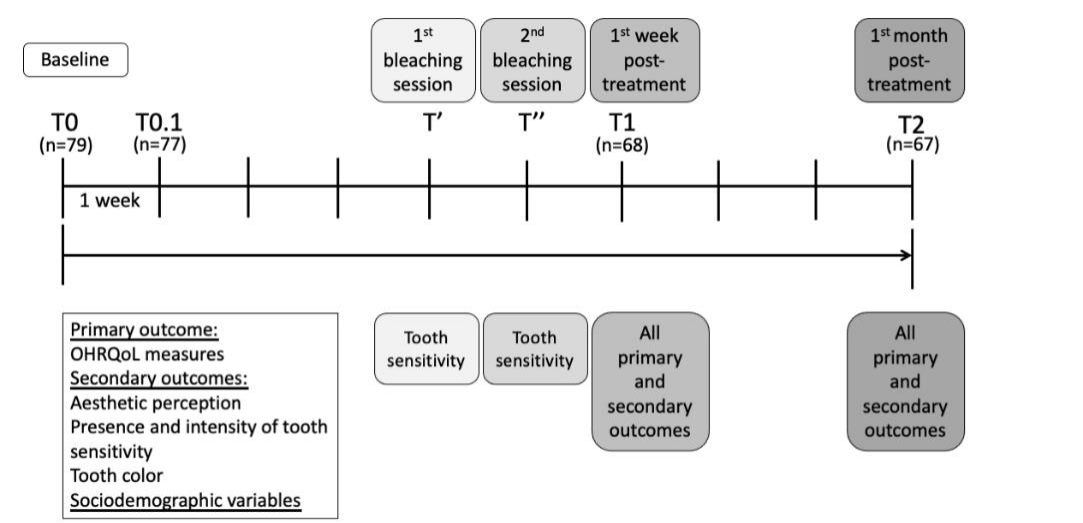
Study timeline, with evaluation times for primary and secondary outcomes.

The OHIP-Aes questionnaire was applied two times before the intervention (time 0 and time 0.1), one week (time 1) and one month (time 2) after the intervention (Figure 1).

### Development of the Portuguese version of OHIP-Aes

**Figure 2:**
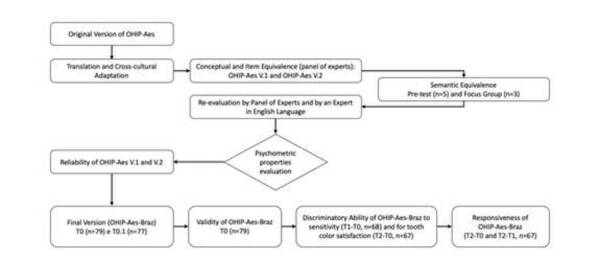
Flowchart of the translation and cross-cultural adaptation and evaluation of the psychometric properties of the instrument.

A flowchart of the translation and cross-cultural adaptation steps and the psychometric properties evaluation of the Portuguese version of OHIP-Aes is presented in Figure 2.

The OHIP-Aes instrument was originally developed and validated in the English language and derived from OHIP-49 items ^8^. It comprises 14 questions covering seven dimensions: functional limitation (items 1 and 2), physical pain (items 3 and 4), psychological discomfort (items 5 and 6), physical disability (items 7 and 8), psychological disability (items 9 and 10), social disability (items 11 and 12) and handicap (items 13 and 14) ^8^.

For each of the 14 questions from OHIP-Aes, patients are asked how often they experienced the impact “in the past two weeks”. The original OHIP-49 investigates impacts in the last six months. This change was proposed and tested in a previous study, in order to be sensitive to changes in OHRQoL of patients undergoing tooth whitening and proved to be adequate ^3^ ^,^ ^8^. The answers, as in the original instrument, were presented in a Likert scale coded as ‘never’ (0), ‘hardly ever’ (1), ‘occasionally’ (2), ‘fairly often’ (3), and ‘very often’ (4) ^3^ ^,^ ^8^ ^,^ ^15^. The total score is calculated by adding the items scores and can vary from 0 to 56. The higher the score, the worse the aesthetic oral health impact.

### Conceptual and item equivalence

For the development of the Portuguese version of OHIP-Aes, the original instrument developed in English language ^8^ was analyzed for conceptual and item equivalence by a panel of experts, using the focus group methodology. This first step aimed to verify whether the items selected for the English language instrument were relevant to the Brazilian culture ^16^ since OHIP-Aes was initially validated in a population of young Chinese adults.

At the end of this stage, the group of experts concluded that some items of OHIP-Aes in English differed from the items that they considered appropriate for the Brazilian culture. Therefore, two versions of the instrument were tested. The first version (named OHIP-Aes-V1) consisted of the translation-back-translation of the 14 items enclosed in the original instrument, published in English. The second version (named OHIP-Aes-V2) consisted of 11 questions translated from the original instrument plus three questions suggested by the panel of experts to be tested as substitutes for questions 1, 2, and 9 of OHIP-Aes-V1. They were chosen from the 49-item original instrument version ^3^: “Did you have difficulty chewing any food because of problems with your teeth, mouth or dentures?”, “Did you have trouble pronouncing a word because of problems with your teeth, mouth or dentures?” and “Were you upset because of problems with your teeth, mouth or dentures?” and translated into Portuguese.

### Semantic equivalence

A pre-test of the questionnaire preliminary version was carried out with a group of individuals with sociodemographic characteristics similar to those who would be part of the main study (young adults of both sexes and who had completed elementary school). Individual interviews followed by a focus group were conducted ^16^. Initially, five people were individually interviewed to express their understanding of the questionnaire and explain how they arrived at their respective answers. The interviews were recorded so that more than one analyst could evaluate them later. Subsequently, three participants were gathered into a focus group in order to discuss the aspects of the questionnaire that were identified as unclear during the individual interviews. They could also suggest terms or adaptations in the items, favoring their understanding, if necessary.

After the pre-test stage, the changes or adaptations suggested in the wording of items were re-evaluated by the panel of experts at the final review of the instrument. An expert in the English language judged whether the suggested changes would help the comprehension of the instrument in the Portuguese language without changing the meaning of the item in the English language.

### Reliability

The reliability of both versions (OHIP-Aes-V1 and OHIP-Aes-V2) was assessed by their stability and internal consistency. The internal consistency was determined by the correlation between different items that compose the instrument and the degree of scale homogeneity. The results of the instrument applied at time 0.1 were analyzed for inter-item correlation and total item-score correlation (item-rest), using Cronbach's alpha coefficient. After that, a final version of the instrument, derived from the tested items that presented better internal consistency, was used for subsequent tests. This version was named OHIP-Aes-Braz.

According to the test-retest model, the OHIP-Aes-Braz instrument stability was determined applying the instrument twice to the same individual, in a time interval in which there was no change in the individual’s state concerning the dental aesthetics. Therefore, the group of 79 individuals seeking bleaching treatment answered the questionnaire at the beginning of the study (baseline). After one week (time 0.1), without any therapeutic intervention or any change in the dental aesthetics, 77 individuals answered the questionnaire again. The agreement of the scores obtained in both moments was evaluated using the intraclass correlation coefficient (ICC).

### Evaluation of the validity of OHIP-Aes-Braz

The validity of OHIP-Aes-Braz, that is, its ability to detect impacts of dental aesthetics on individuals’ quality of life, was assessed by the association between the total score of the instrument and clinical and subjective parameters related to dental aesthetics, both measured at baseline (time 0).

The convergent construct validity was evaluated by the correlation between the total score of the instrument and the aesthetic self-perception single item question. The question used was: “How much are you satisfied with the color of your teeth?”. The responses were recorded in a five-point Likert scale: ‘very dissatisfied’ ^1^, ‘dissatisfied’ ^2^, ‘neither satisfied nor dissatisfied’ ^3^, ‘satisfied’ ^4^, and ‘very satisfied’ ^5^. Convergent validity was also assessed through the correlation between the total score of the instrument and the individuals’ oral health self-perception, which was also measured in an ordinal scale ranging from 1 to 5. The higher the score, the better the oral health perception ^15^.

The concurrent criterion validity was assessed by the correlation between the total scores of OHIP-Aes-Braz and the total scores of the validated Brazilian versions of the instruments OHIP-14 ^15^ and Oral Impact on Daily Performances (OIDP) ^17^. The OIDP has already been used to measure impacts related to tooth whitening on Brazilian individuals’ OHRQoL ^18^.

### Evaluation of the discriminatory ability of OHIP-Aes-Braz

 The OHIP-Aes-Braz discriminatory ability was analyzed by comparing the differences between the total scores after tooth whitening in patients with and without reported sensitivity one week post-treatment (time 1), and in dissatisfied/neutral patients and patients satisfied with the color of their teeth one month after treatment (time 2). The Wilcoxon test was used.

### Responsiveness of OHIP-Aes-Braz

The OHIP-Aes-Braz responsiveness, that is, its ability to detect changes over time resulting from the intervention, was assessed by sensitivity to change in the construct in patients undergoing tooth whitening. The OHIP-Aes-Braz instrument sensitivity in detecting these changes was evaluated by the difference between the total scores before (time 0) and after treatment (time 2).

The responsiveness of OHIP-Aes-Braz was also compared to the changes detected using OHIP-14 and OIDP, through the correlation of the differences between the final and initial scores of the instruments (() and the post-bleaching color of the upper central incisors.

The comparison of pre- and post-treatment scores was performed using the paired Wilcoxon test. Correlations were assessed using Spearman correlation coefficient. The software Stata SE, version 15.0 (StataCorp LCC, College Station TX, USA), was used for all analyzes, adopting a 5% significance level.

## Results

One hundred twenty-one patients were examined for eligibility. Seventy-nine patients were included in the randomized clinical trial. The sociodemographic characteristics of the study population were as follows: 63.3% were female, the mean age was 24.4 + 5.0 years old, the marital status was single for 83.5%, the mean years of schooling was 15.3 + 1.7, and 64.6% had monthly family income of more than five Brazilian minimum wages (1BMW=US$ 193.00 at the study time). Sixty-seven patients were evaluated at the end of follow-up (time 2), considering losses to follow-up (n=11) and one patient who did not receive the intervention for declining to participate after inclusion in the study.

### Reliability assessment of the OHIP-Aes-V1 and OHIP-Aes-V2 tested versions

Although Cronbach's alpha coefficient was high for both versions, items 1 and 2 of the OHIP-Aes-V1 version and item 9 of the OHIP-Aes-V2 version contributed to a better internal consistency, that is, a better correlation between items and the total score.

Thus, the final tested version included the 13 questions translated from the original instrument ^8^, the OHIP-Aes-V1, and one question (item 9) extracted from the panel of experts’ recommendations. This final version of the instrument was named OHIP-Aes-Braz and it was used for the stability and internal consistency analysis, as well as for all other analysis of validity, discriminatory ability and responsiveness. The instrument in the Portuguese language is shown in Appendix A. The Cronbach’s alpha coefficient for OHIP-Aes-Braz total score was 0.92 (Table 1). For test-retest reliability, the ICC was 0.95 (95%CI 0.92-0.97).

**Table 1:** Internal consistency for the total score and items of the OHIP-Aes-Braz.

Item	Item-test correlation	Item-rest correlation	Cronbach’s alpha*
OHIP-Aes-Braz 1	0.7937	0.7314	0.9127
OHIP-Aes-Braz 2	0.8602	0.8137	0.9090
OHIP-Aes-Braz 3	0.2615	0.1592	0.9314
OHIP-Aes-Braz 4	0.2214	0.1302	0.9304
OHIP-Aes-Braz 5	0.8733	0.8448	0.9077
OHIP-Aes-Braz 6	0.8761	0.8397	0.9071
OHIP-Aes-Braz 7	0.4674	0.4412	0.9224
OHIP-Aes-Braz 8	0.943	0.9261	0.9031
OHIP-Aes-Braz 9	0.8445	0.8106	0.9089
OHIP-Aes-Braz 10	0.9277	0.9085	0.9044
OHIP-Aes-Braz 11	0.6691	0.6463	0.9192
OHIP-Aes-Braz 12	0.7510	0.7302	0.9172
OHIP-Aes-Braz 13	0.8092	0.7757	0.9112
OHIP-Aes-Braz 14	0.8204	0.8008	0.9144
Total Score			0.9205

### Assessment of the OHIP-Aes-Braz validity

The OHIP-Aes-Braz instrument showed convergent construct validity since the baseline total scores presented a significant negative correlation with color satisfaction and self-perception of oral health, as expected (Table 2). Individuals less satisfied with the color of their teeth reported a greater impact on OHRQoL, and the better the individual’s self-perceived oral health, the lower the instrument total scores.

**Table 2:** Convergent and concurrent criterion construct validity assessment.

	Convergent validity				Concurrent criterion validity			
	Satisfaction with tooth color		Self-perceived oral health		OHIP-14 total score		OIDP total score	
	*r*_s_	p-value	*r*_s_	p-value	*r*_s_	p-value	*r*_s_	p-value
OHIP-Aes-Braz total score	-0.25	0.03	-0.50	0.000	0.63	<0.001	0.77	<0.001

*r*_s_, Spearman's rank correlation coefficient

When evaluating the concurrent criterion validity through the correlation between the OHIP-Aes-Braz and OHIP-14 and OIDP total scores, it was observed that there were significant positive correlations between the instruments. The greater the impact measured by OHIP-Aes-Braz, the greater the impact measured by OHIP-14 and OIDP (Table 2).

### Discriminatory ability

When comparing the difference in post-whitening total scores in dissatisfied/neutral patients and patients satisfied with tooth color at the end of treatment, OHIP-Aes-Braz was able to discriminate these groups of patients (Mann-Whitney test, p = 0.01).

The ability of OHIP-Aes-Braz to discriminate individuals with and without tooth sensitivity was assessed at the immediate postoperative follow-up (time 1, one week after the last tooth whitening session). At this time, the postoperative sensitivity was still elevated. The mean total score of the instrument in patients without sensitivity (2.93 ± 4.8) was lower than in patients with sensitivity (4.13 ± 6.0); however, this difference was not statistically significant (Mann-Whitney test, p = 0.12). It is worth mentioning that 30 days after the end of the treatment no individuals reported tooth sensitivity (data not shown).

### Responsiveness

**Figure 3:**
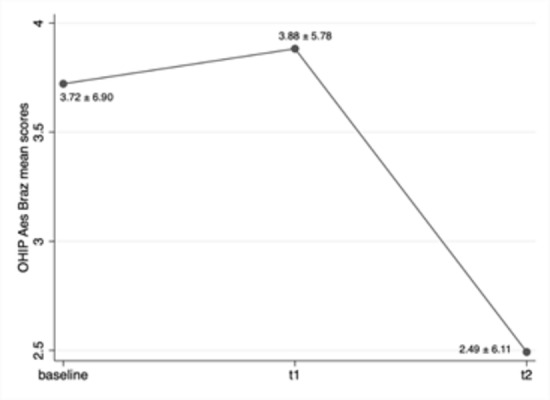
Variation in the mean of OHIP-Aes-Braz total scores at different study times.

There was a significant difference in OHIP-Aes-Braz total scores between times 0 and 2 (paired Wilcoxon test, p<0.001) and also between times 1 and 2 (paired Wilcoxon test, p <0.001) (Figure 3), demonstrating that the instrument was sensitive to detect changes in OHRQoL resulting from an aesthetic intervention.

When comparing the OHIP-Aes-Braz responsiveness to the changes regarding the clinical difference in the upper central incisors color using OHIP-14 (*r*_s_=0.01, p=0.94) and OIDP (*r*_s_=-0.04, p=0.74), it was observed that only the variation in the OHIP-Aes total score was statistically significant (*r*_s_=-0.26, p=0.03). It means that OHIP-Aes-Braz was responsive to clinical outcomes related to the dental aesthetics, that is, the bleached teeth color, whereas OIDP and OHIP-14 were not.

## Discussion

The psychometric properties of Portuguese version of OHIP-Aes-Braz proved to be adequate, considering the validity and the excellent stability and internal consistency observed. The final version of the instrument validated in this study, OHIP-Aes-Braz, derivated from the original instrument OHIP-Aes published in English and validated in a Chinese population ^8^. We replaced an item (item 9 in the OHIP-Aes original version) by another (item 34 in the original OHIP-49) as suggested by the expert panel. The original question, “Have you got difficult to relax because of problems with your teeth, mouth or dentures?” was replaced by “Have you got upset because of problems with your teeth, mouth or dentures?”. Considering the results of the statistical analyses, it is reasonable to believe that this approach did not compromise the validity of the final Brazilian version of the instrument. Actually, the questionnaire containing this item proved to be more appropriate to represent the construct of interest in the studied population.

The proposed instrument, OHIP-Aes-Braz, showed excellent stability and internal consistency, and a moderate correlation among almost all items. The temporal stability was equivalent to that assessed on the other two validated versions of the OHIP-Aes (5, 8). Although an internal consistency greater than 0.70 is considered satisfactory for comparisons between groups ^19^, the internal consistency in the present study was higher than that assessed on the English language validation ^8^ and in the Spanish language ^5^.

When assessing instrument’s homogeneity, moderate correlations between items are desirable; high correlations may reveal redundancy and low correlations imply that they do not offer a relevant contribution to the scale. Items 3 (sensitivity to cold and hot foods) and 4 (pain in the oral cavity) showed values of correlation with the total scale score below 0.20, which might suggest that they could be deleted since, according to Streiner and Norman ^20^, an item-rest correlation below 0.20 indicates the need to remove or reformulate these items. However, before eliminating items with low correlation, it is prudent to consider the rationale behind the development of the items; low values of correlation between some items and the total score were also observed in other studies that justified the maintenance of these items ^21^.

The OHIP-Aes-Braz instrument items assess more than one construct dimension so that weak correlations between items and the total score may occur. In the present study, both items that showed a lower correlation with the scale total score belong to the “physical pain” domain. A plausible explanation would be the sample’s socioeconomic characterization, which could probably justify a greater use of dental services and the absence of pain and sensitivity before clinical intervention (when the instrument internal consistency was assessed). On the other hand, aesthetic interventions can cause transient postoperative sensitivity or pain, such as post-bleaching sensitivity, so that these individuals’ quality of life can be modulated for better or worse depending on the balance between aesthetic improvement and side effects of treatment ^22^. Thus, the presence of items related to the “physical pain” dimension is relevant in instruments that can be used to assess OHRQoL in aesthetic clinical intervention studies.

In addition to presenting excellent stability and internal consistency, the OHIP-Aes-Braz instrument showed construct and criterion validity. There was a significant negative correlation between OHIP-Aes-Braz and satisfaction with tooth color, as well as with individuals’ self-perceived oral health. It means that individuals less satisfied with their tooth color had a greater negative impact on OHRQoL and those with better self-perceived oral health had a lesser negative impact on their quality of life. For criterion validity, OHIP-Aes-Braz measurements were in the same direction as the other instruments tested, OIDP and OHIP-14.

The OHIP-Aes-Braz was also able to discriminate satisfied patients from those not satisfied with tooth color at the post-treatment, similar to versions in other languages ^5^ ^,^ ^8^. The instrument was also able to detect changes due to an aesthetic intervention, proving to be responsive. The OHIP-Aes-Braz was responsive for clinical outcomes related to dental aesthetics (whitened teeth) whereas OIDP and OHIP-14 were not. The positive change in quality of life of patients who had their teeth whitened was evident in the dental aesthetics self-perception one month after treatment, which supports the hypothesis that tooth whitening positively impacts the individuals’ OHRQoL and improves the dental aesthetics self-perception at the late postoperative period.

It is expected that once the tooth whitening effect persists, patients will continue to experience positive psychological, social and functional impacts ^11^. Estay et al. ^12^ showed that tooth whitening was associated with more favorable attitudes towards oral health and a better self-perceived image, with immediate and long-term social, psychological, and aesthetic positive impacts. However, this hypothesis deserves future studies.

It is important to clarify that this study was primarily designed to test the efficacy of dental bleaching on a sample of adults living in a Brazilian city. The sample consisted mostly of young individuals who sought an exclusively aesthetic treatment, with family income above five minimum wages and who had attended an average of 15 years of study. It should not be considered a representative sample of the Brazilian population. Then, the generalization of our results must be made with caution; testing the instrument before using it in other settings is recommended. Furthermore, other aspects deserve to be pointed out as possible limitations of the study. There was a dropout rate of 15% which could have resulted in selection bias. However, the characteristics related to sociodemographic and clinical and subjective measures were similar between those who discontinued and those who were followed until the end of the study. In addition, the study power was not compromised, since it was within the rate calculated at the beginning of the study, satisfying the minimum sample of patients.

Another relevant issue is that the color change was the main clinical aspect investigated. Although tooth color is one of the main characteristics concerning dental aesthetics, it would be suitable to test this instrument in other interventions intended to modify other important aspects of dental aesthetic, such as the size, shape, and position of teeth ^3^. The original reduced version of OHIP, OHIP-14, has been extensively used in clinical studies for various aesthetic interventions such as prosthetic rehabilitation ^23^, implants ^24^, and orthodontic treatment ^25^. It would be useful to test if OHIP-Aes, compared to OHIP-14, would have better psychometric properties or be more responsive to these aesthetic interventions, just as it was in this study for tooth whitening.

The Portuguese version of the OHIP-Aes instrument, OHIP-Aes-Braz, which was adapted and tested in this study showed reliability, in terms of internal consistency and stability. It also proved to be valid, discriminatory, and responsive, being sensitive to the change in the perception of OHRQoL in adults submitted to tooth bleaching.

## References

[1] Glick M, Williams DM, Kleinman DV, Vujicic M, Watt RG, Weyant RJ (2016). A new definition for oral health developed by the FDI World
Dental Federation opens the door to a universal definition of oral
health. Br Dent J.

[2] Locker D, Allen F (2007). What do measures of ‘oral health-related quality of life’
measure?. Community Dent Oral Epidemiol.

[3] McGrath C, Wong AHH, Cheung CS (2005). The sensitivity and responsiveness of an oral health related
quality of life measure to tooth whitening. J Dent.

[4] Bersezio C, Martín J, Herrera A, Loguercio A, Fernández E (2018). The effects of at-home whitening on patients’ oral health,
psychology, and aesthetic perception. BMC Oral Health.

[5] Núñez L, Dreyer E, Martín J, Moncada G (2013). Validation of the Spanish OHIP-Aesthetic questionnaire for
Chilean adults. J Dent Oral Craniofac Epidemiol.

[6] Bruhn AM, Darby ML, McCombs GB, Lynch CM (2012). Vital tooth whitening effects on oral health-related quality of
life in older adults. J Dent Hyg.

[7] Martin J, Vildosola P, Bersezio C, Herrera A, Bortolatto J, Saad JRC (2015). Effectiveness of 6% hydrogen peroxide concentration for
toothbleaching - a double-blind, randomized clinical trial. J Dent.

[8] Wong AHH, Cheung CS, McGrath C (2007). Developing a short form of Oral Health Impact Profile (OHIP) for
dental aesthetics: OHIP-aesthetic. Community Dent Oral Epidemiol.

[9] Kothari S, Gray AR, Lyons K, Tan XW, Brunton PA (2019). Vital bleaching and oral-health-related quality of life in
adults: A systematic review and meta-analysis. J Dent.

[10] Bersezio C, Martín J, Peña F, Rubio M, Estay J, Vernal R (2017). Effectiveness and impact of the walking bleach technique on
esthetic self-perception and psychosocial factors: A randomized double-blind
clinical trial. Oper Dent.

[11] Bersezio C, Martín J, Angel P, Bottner J, Godoy I, Avalos F (2019). Teeth whitening with 6% hydrogen peroxide and its impact on
quality of life: 2 years of follow-up. Odontology.

[12] Estay J, Angel P, Bersezio C, Tonetto M, Jorquera G, Peña M (2020). The change of teeth color, whiteness variations and its
psychosocial and self-perception effects when using low vs. high
concentration bleaching gels: a one-year follow-up. BMC Oral Health.

[13] Fernández E, Bersezio C, Bottner J, Avalos F, Godoy I, Inda D (2017). Longevity, Esthetic Perception, and Psychosocial Impact of Teeth
Bleaching by Low (6%) Hydrogen Peroxide Concentration for In-office
Treatment: A Randomized Clinical Trial. Oper Dent.

[14] Locker D, Jokovic A, Clarke M (2004). Assessing the responsiveness of measures of oral health-related
quality of life. Community Dent Oral Epidemiol.

[15] Oliveira BH, Nadanovsky P (2015). Psychometric properties of the Brazilian version of the Oral
Health Impact Profile - Short form. Community Dent Oral Epidemiol.

[16] Leão AT, Oliveira BH, Luiz RR, Costa AJ, Nadanovsky P (2005). Epidemiologia e bioestatística na pesquisa odontológica.

[17] Cortes MIS, Marcenes W, Sheiham A (2002). Impact of traumatic injuries to the permanent teeth on the oral
health-related quality of life in 12-14-year-old children. Community Dent Oral Epidemiol.

[18] Meireles SS, Goettems ML, Dantas RV, Bona AD, Santos IS, Demarco FF (2014). Changes in oral health related quality of life after dental
bleaching in a double-blind randomized clinical trial. J Dent.

[19] Bland JM, Altman DG (1997). Statistics notes - Cronbach’s alpha. BMJ.

[20] Streiner D, Norman G (1995). Health measurement scales: a practical guide to their development and
use.

[21] Cohen-Carneiro F, Rebelo MAB, Souza-Santos R, Ambrosano GMB, Salino AV, Pontes DG (2010). Psychometric properties of the OHIP-14 and prevalence and
severity of oral health impacts in a rural riverine population in Amazonas
State, Brazil. Cad Saúde Pública.

[22] Goettems ML, Fernandez M, Donassollo TA, Donassollo SH, Demarco FF (2020). Impact of tooth bleaching on oral health-related quality of life
in adults: A triple-blind randomised clinical trial. J Dent.

[23] Øzhayat EB, Gotfredsen K (2019). Patient-reported effect of oral rehabilitation. J Oral Rehabil.

[24] Toia M, Wennerberg A, Torrisi P, Farina V, Corrà E, Cecchinato D (2019). Patient satisfaction and clinical outcomes in implant-supported
overdentures retained by milled bars: two-year follow-up. J Oral Rehabil.

[25] Feu D, Miguel JA, Celeste RK, Oliveira BH (2013). Effect of orthodontic treatment on oral health-related quality of
life. Angle Orthod.

